# Development of a Framework for On-Demand Caesarean Section in Romania

**DOI:** 10.3390/ijerph20032705

**Published:** 2023-02-02

**Authors:** Ion Petre, Flavia Barna, Cosmin Cîtu, Florin Gorun, Oana-Maria Gorun, Laurentiu Cezar Tomescu, Adrian Apostol, Anca Bordianu, Cristian Furau, Izabella Petre

**Affiliations:** 1 Department of Functional Sciences, Medical Informatics and Biostatistics Discipline, “Victor Babes” University of Medicine and Pharmacy Timisoara, 2 Eftimie Murgu Square, 300041 Timisoara, Romania; 2 Doctoral School, Faculty of Economics and Business Administration, West University of Timisoara, 16 J. H. Pestalozzi Street, 300115 Timisoara, Romania; 3 Department of Finance, Faculty of Economics and Business Administration, West University of Timisoara, 16 J. H. Pestalozzi Street, 300115 Timisoara, Romania; 4 Department of Obstetrics and Gynecology, “Victor Babes” University of Medicine and Pharmacy Timisoara, 2 Eftimie Murgu Square, 300041 Timisoara, Romania; 5 Department of Obstetrics and Gynecology, Municipal Emergency Clinical Hospital Timisoara, 1-3 Alexandru Odobescu Street, 300202 Timisoara, Romania; 6 Doctoral School, “Victor Babes” University of Medicine and Pharmacy Timisoara, 2 Eftimie Murgu Square, 300041 Timisoara, Romania; 7 Faculty of Medicine, Ovidius University of Constanta, 900527 Constanta, Romania; 8 Department VII of Internal Medicine, “Victor Babes” University of Medicine and Pharmacy Timisoara, 2 Eftimie Murgu Square, 300041 Timisoara, Romania; 9 Departament of Plastic Surgery and Reconstructive Microsurgery Bagdasar-Arseni, Emergency Hospital Bucharest, 014461 Bucharest, Romania; 10 Department of Obstetrics and Gynecology, Faculty of Medicine, “Vasile Goldiș” Western University of Arad, 310025 Arad, Romania

**Keywords:** c-section, budget-impact, economic analysis

## Abstract

Background: Caesarean section rates have continued to trend upward in most countries, including Romania, creating a number of economic challenges. In the public health system, there is no regulation for performing Caesarean sections on demand; it is often done unlawfully, and in private hospitals, it is a real business. Thus, this study aims to investigate the budgetary impact at a hospital level and the profit per procedure by introducing on-demand caesarean sections for a fee. Methods: This study was conducted in one of the largest maternity units in Western Romania—the “Bega” Maternity Clinic of the Timisoara County Emergency Hospital. For the analysis, the difference between a proposed occupancy rate (between 50 and 85%, increasing every 5 percent) and the actual occupancy rate was calculated. Considering that this difference can be used to admit patients to receive Caesarean sections on demand for a fee, the profit that could be obtained during the study period was calculated. Results: It is reported that between 238 (proposed occupancy rate of 50%) and 4683 patients (a proposed occupancy rate of 85%) could have benefited from on-demand caesarean section surgery in 2017–2019. Between RON 419,999 and RON 8,551,636 could be obtained in the 3 years of study by implementing caesarean section against payment. Conclusion: The implementation of a system of on-demand payment for caesarean sections in Romania would bring significant profits to the hospital budget.

## 1. Introduction

Caesarean birth (C-section) is a leading benchmark for access to vital, life-saving obstetric care. A C-section can reliably prevent maternal and perinatal mortality and morbidity where it is medically justified. The C-section has also become a significant indicator of progress in emergency obstetric care and a means of avoiding complications during delivery and labour [1]. Furthermore, very low C-section rates (less than 15%) are associated with worse outcomes for both mother and infant [2]. C-section rates have continued to trend upward in the majority of industrialised countries, creating a variety of economic challenges [3]. The global C-section rate was reported to be 21.1%, with averages between 8.2 and 27.2% depending on the region [3]. These rates have increased in recent decades from around 7% in 1990, and this trend is predicted to continue into the current decade. By 2030, according to some research, almost 29% of all births worldwide will be by caesarean section [4]. The countries with the highest C-section rate worldwide were the Dominican Republic with 58.1%, Brazil with 55.7%, Cyprus with 55.3%, Egypt with 51.8%, and Turkey with 50.8%. In Europe, the caesarean rate is 39.3% in Poland, 16.6% in Sweden, 19.7% in France, 33.6% in Italy, and 30.1% in Germany, with the lowest being in Finland (16.5%). In Romania, the C-section rate is one of the highest on the European continent, second only to Cyprus, being reported in 2017 at 44.1% [5].

However, these changes in birth patterns can have substantial economic implications. In Romania, the National Health Insurance House (CNAS) pays hospitals up to RON 3400 (EUR 692) for a caesarean section, compared to RON 1400 (EUR 285) for a natural birth without complications. In 2019, the median cost of a caesarean section was USD 11,326 in the United States, USD 7948 in Switzerland, and USD 3704 in Germany, while the median cost of a vaginal birth in these countries was USD 7500, USD 5634, and USD 2448, respectively [6,7]. Furthermore, a study carried out in Ireland and England/Wales found that if both regions were to achieve CS rates that are at the same level as the best performing countries in Europe, the net current value of additional cost savings is estimated between EUR 3.5 million and GBP 23.0 million, respectively [8]. Moreover, even though caesarean section can be a life-saving operation for a foetus at risk, paradoxically, countries with higher rates of C-section have consistently higher rates of neonatal morbidity and mortality. Furthermore, the risks of haemorrhage, sepsis, venous thromboembolism, and amniotic fluid embolism are approximately five times higher with a C-section compared to a vaginal birth [9]. Additionally, it was reported that patients who underwent caesarean sections had a 9% lower subsequent pregnancy rate and an 11% lower birth rate compared to patients who delivered vaginally [10].

A Caesarean section on demand can be defined as a primary C-section delivery at the request of the mother in the absence of any maternal or foetal indication [11]. Several guidelines, such as those of “The American College of Obstetricians and Gynecologists” (ACOG), “The National Institute for Health and Care Excellence” (NICE), “The Royal Australian and New Zealand College of Obstetricians and Gynaecologists” (RANZCOG), and the Italian National Institute of Health (ISS), support caesarean section at the mother’s request, provided women are adequately informed [12]. Requests for an on-demand C-section are increasingly found in clinical practice worldwide, representing a medical, financial, and ethical dilemma [13]. In addition, financial incentives may encourage private providers to perform more caesarean sections than non-profit hospitals [14]. Caesarean sections are big business in Romania’s private healthcare system. 

In public hospitals, there are guidelines and protocols that refer to recommendations about situations that require caesarean birth. Also, a caesarean section is by no means recommended as a more comfortable option, to the detriment of natural delivery.

This study aims to investigate the budget impact at the individual hospital level and the profit per procedure by introducing on-demand caesarean sections.

## 2. Materials and Methods

### 2.1. Target Population

The eligible population was women of reproductive age residing in one of the 5 counties in the western area of Romania (Timis County, Caras-Severin County, Arad County, Hunedoara County, and Bihor County). This region of Romania includes 443,473 women aged 14–49. The largest county in this region, and even in the country, is Timis County, with its administrative capital in Timisoara. The number of live births in Timis County was 6689 in 2020, 6808 in 2021, and 4272 until September 2022. In 2020, the fertility rate in Romania was 1.8 times higher than in the European Union, where it was 1.5 [15].

This study has been approved by the Ethics Committee of the Clinical Emergency Hospital “Pius Brinzeu” Timisoara (No. 95/28 October, 2019).

### 2.2. Study Settings

This study was conducted in one of the largest maternity units in Western Romania—the “Bega” Maternity Clinic of the Timisoara County Emergency Hospital. Situated in the largest city in the western region of Romania, in Timisoara, the “Bega” Maternity Hospital is an integrated part of the largest hospital in western Romania—Timisoara Emergency County Clinical Hospital. The “Bega” Maternity Hospital is administratively divided into two wards, namely: Obstetrics-Gynaecology Ward I (OG1) and Obstetrics-Gynaecology Ward II (OG2). There is no administrative difference between the two sections (OGI and OGII), both having the same capacity. Both wards are divided into several sectors; this study was carried out only on the “delivery block” sector, where patients are admitted for delivery. The occupancy rate estimates were also carried out, taking into account only this sector, which includes the postnatal care areas.

The current study assessed the situation of the number of cases in the period 2017–2019, with the year 2020 not being relevant due to the pandemic and the fact that “Bega” Maternity Hospital has been declared a Maternity Hospital COVID-19 support—only patients confirmed to have SARS-CoV2 infection were admitted.

### 2.3. The Romanian Context

The Romanian social health insurance system covered 89% of the population in 2017 and proposes to ensure universal health insurance coverage. The Romanian Ministry of Health is responsible for overall governance, while the National Health Insurance House (CNAS) manages and regulates the system. The health system in Romania is mainly financed from four different sources: the national health insurance funds, the state budget, local budgets, and out-of-pocket payments [15].

The health system in Romania allows patients to choose their doctor without any additional cost. This can lead to discrepancies between the occupancy rates of wards.

### 2.4. Measurement and Valuation of Resources and Costs

For the analysis, the difference between a proposed occupancy rate (between 50 and 85%, increasing every 5 percent) and the actual occupancy rate was calculated. This difference is related to an average hospitalisation time of 4 days/patient, resulting in the number of patients who could benefit from paid medical services in the 2 wards of the “Bega” Maternity Clinic of the Timisoara County Emergency Hospital.

The profit estimate has been calculated in all variants (proposed occupancy from 5 to 85%), taking into account that the difference between the proposed and current occupancy levels will be dedicated only to women who will be provided with Caesarean sections on-demand. Several variants of the proposed occupancy rate (every 5 percent) were taken to demonstrate the profit in several situations, as it is not clear how many patients will request this service.

The proposed maximum occupancy level of 85% was chosen in order to reserve vacant beds in the ward for unexpected situations.

### 2.5. Outcomes

The primary outcome was the profit achieved in the two Obstetrics and Gynecology wards following the implementation of the on-demand caesarean section service for a fee.

### 2.6. Currency, Price Date, and Conversion

The amounts were reported in the national currency of Romania, namely the Romanian leu (RON), as well as in euros (EUR). The RON-EUR conversion was carried out at the National Bank of Romania’s average exchange rate for 2022 (EUR 1 = RON 49,321).

The annual expenditure reports of the OG1 and OG2 wards of the Clinical Emergency Hospital “Pius Brinzeu” Timisoara (SCJUPBT) were used to determine the costs of deliveries. Thus, the average cost for a natural childbirth is RON 3532 case (EUR 716.12 case) and RON 5166 case (EUR 1047.42 case) for a caesarean section. The proposed additional fee for the surgical team, according to the rates charged by private clinics, is RON 3500 case (EUR 709.63 case) per caesarean section. The proposed unit prices for fee-based medical services are similar to those in private clinics (90%), at RON 12,000 (EUR 2433) for caesarean sections (Table 1).

## 3. Results

### 3.1. Current Occupancy Rate of the Maternity Wards

Between 2017 and 2019, 7920 births took place in the maternity ward, with the most in 2017 (2608 births) (Table 1).

It should be recalled that, for the purposes of the analysis, the difference between a proposed occupancy rate (between 50 and 85%, increasing every 5 percent) and the actual occupancy rate was calculated. In the OGI ward between 2017 and 2021, the maximum occupancy rate was 76.4% (year 2017), while in the OGII ward, the maximum occupancy rate was 47.9% (year 2019). The difference bridging between a proposed occupancy rate and the current occupancy is shown in Table 2.

It is reported that between 238 (a proposed occupancy rate of 50%) and 4683 patients (proposed occupancy rate of 85%) could have benefited from on-demand caesarean section surgery in 2017–2019 (Table 3).

### 3.2. Budget Impact Analysis

The net additional income that can be obtained by implementing the on-demand healthcare payment system is shown in Table 4 and Table 5, detailed for the three years of study, for each ward, based on a proposed occupancy rate in the range of 50–85%, with analysis for each 5% step.

## 4. Discussion

Health budgets and the way the health system is financed are key determinants of the health of a country’s population. Romania has one of the lowest health expenditures as a share of gross domestic product (GDP) among European Union (EU) member states (5.16%) [16]. Romania also has one of the highest rates of caesarean sections in Europe; many of these are performed at the request of the mother, even if this is not regulated. Thus, this study aimed to highlight the budgetary impact of implementing a payment system for on-demand C-sections.

In our sample, the caesarean section rate was between 53.6 and 60.7%, many of which were performed on request even if this was not declared. Romanian legislation does not regulate the conditions under which such a procedure can be performed at the mother’s request. However, in order to reduce the number of unnecessary caesarean sections, it introduced the co-payment system for caesarean sections on demand, but this is still not implemented. Another interesting result from this study was the substantial difference between the current occupancy rates between the two departments of the same hospital. This may be due to the fact that the legislation in Romania allows the patient to choose his physician without additional payment.

In Romania, the responsibility for the overall governance of social health insurance (SHI) is provided by the Ministry of Health, part of the National Government, while the national health insurance system is administered by the National Health Insurance House governing the Insurance Fund (NHIF). At the local level, the Ministry as well as the National Health Insurance House are represented through district public health authorities. Under the SHI system, district health insurance funds purchase services from health service providers at the local level.

In addition, health service providers are paid by the Ministry of Health under national health programmes that cover priority areas such as maternal and child health.

In recent years, Romania has significantly increased its level of health funding, but it remains one of the EU countries with the lowest health spending per capita and as a share of gross domestic product (GDP).

Hospitals receive prospective payments, which are a combination of payment methods. The total amount of the hospital contract is composed of: diagnosis-related groups (DRG), case payments, day rates, a fixed amount dedicated to national curative public health programs, and free-for-service (FFS) payments for services provided by outpatient departments [17]. Emergency services, such as emergency caesarean sections, are paid for from the state budget.

A high number of caesarean operations will put financial pressure on the National Health System. To reduce the number of unnecessary caesarean sections, Romania has recently introduced a co-payment system for on-demand C-sections, but this system remains to be implemented [18].

One of the most important ethical dilemmas in the decision-making process regarding the choice of Caesarean section delivery method concerns the correct understanding of informed consent, which was introduced in Romania in 2003. Under law 95/2006 on the reform of the healthcare system, which stipulates that patients over 18 years of age are obliged to sign a consent form, the patient has the right to be informed about the risks associated with clinical investigations and treatments [18,19]. The Caesarean section was introduced as a failure of natural childbirth due to special obstetrical conditions, but due to the fear of legal repercussions that could result from complications during labour, physicians advised a caesarean section even in the absence of medical indications [18]. A long-standing problem in medicine is that doctors’ fears of malpractice lead to defensive medicine, with caesarean section rates thought to be influenced specifically by fears of malpractice liability [20]. In addition, financial incentives have an important effect on the likelihood of a caesarean section [21]. Satisfaction of medical staff is associated with professional bonuses, with salary increases in the Romanian healthcare system having a positive effect on physician motivation [22].

In low-risk populations elective caesarean sections appear to be more expensive than vaginal birth [3]. However, in Romania, performing caesarean sections on demand is a business in the private healthcare system. The price of such a procedure is over EUR 2000 in most private maternity hospitals, and part of the profit goes to the obstetrician. However, it is not unexpected that the percentage of women giving birth in some clinics and hospitals in Romania has reached 80%, given that the average household income has increased substantially.

The calculations for determining the additional net income that can be obtained by providing medical services—a caesarean section—took into account the possibility of providing medical services by the Emergency County Clinical Hospital, Pius Brînzeu” Timisoara, paid by patients, who can thus benefit from all the facilities offered by the private system in a state clinic, additionally benefiting from the services of other specialties in case of medical complications.

When opting for an occupancy rate of 85%, in order to ensure administrative activity in optimal conditions, the estimated additional annual net income, during the study period 2017–2019, is: RON 1.41 million—RON 1.87 million for the OG1 department, respectively, and RON 3.93 million—RON 4.08 million for the OG2 department.

This paper has several limitations. First, the study has a retrospective design, and the data were obtained from a single clinic. Secondly, considering that this study was carried out in a tertiary clinic, these results need confirmation by highlighting some data from other lower-level hospitals. In addition, it is not known exactly how many patients would choose a caesarean section on demand if there were a fee. Additional survey-based studies would be necessary to implement such a system.

## 5. Conclusions

The birth rate is one of the most important components of a country’s development. However, in order to achieve a birth rate comparable to those existing in developed countries, it is imperative to reach a percentage of GDP allocated to health expenditure comparable to theirs, which is between 7 and 8% if we look at things at a macroeconomic level, and at the level of each state health institution with beds, the manager’s interest should be directed towards creating additional sources of funding. The implementation of a system of on-demand payment for caesarean sections in Romania would bring significant profits to the hospital budget.

## Figures and Tables

**Table 1:** Distribution of cases in “Bega” Maternity Hospital by type of birth.

Year	Natural Delivery Cases		C-Section Cases		Total Births
	OGI	OGII	OGI	OGII	
2017	672	352	1117	467	2608
2018	700	358	1111	487	2656
2019	778	454	998	426	2656
2020	439	271	559	260	1529

OGI = Obstetrics-Gynecology Ward 1; OGII = Obstetrics-Gynecology Ward 2.

**Table 2:** Actual occupancy of wards and difference from planned occupancy.

Year	Ward	Effective Utilisation Rate	Difference up to:							
			85%	80%	75%	70%	65%	60%	55%	50%
2017	OGI	76.38%	8.62	3.62	-	-	-	-	-	-
	OGII	46.51%	38.49	33.49	28.49	23.49	18.49	13.49	8.49	3.49
2018	OGI	74.79%	10.21	5.21	0.21	-	-	-	-	-
	OGII	47.42%	37.58	32.58	27.58	22.58	17.58	12.58	7.58	2.58
2019	OGI	73.59%	11.41	6.41	1.41	-	-	-	-	-
	OGII	47.93%	37.07	32.07	27.07	22.07	17.07	12.07	7.07	2.07

**Table 3:** Potential patients covered by fee-for-service medical services according to the ward level of occupancy.

Year	Ward	Patients Potentially Covered by Fee-For-Service Healthcare (No.)							
		85%	80%	75%	70%	65%	60%	55%	50%
2017	OGI	393	165						
	OGII	1124	978	832	686	540	394	248	102
2018	OGI	466	238	10					
	OGII	1097	951	805	659	513	367	221	75
2019	OGI	521	292	64					
	OGII	1082	936	790	644	498	352	206	60
Total		4683	3561	2502	1990	1552	1114	676	238

**Table 4:** Evolution of assumed net income in RON by ward occupancy from 2017 to 2019 for elective caesarean section births for a fee.

Year	Ward	The Proposed Occupancy Rate							
		85%	80%	75%	70%	65%	60%	55%	50%
2017	OGI	773,620	324,885	-	-	-	-	-	-
	OGII	1,985,968	1,727,983	1,469,998	1,212,013	954,028	696,043	438,058	180,073
	Total	2,759,588	2,052,868	1,469,998	1,212,013	954,028	696,043	438,058	180,073
2018	OGI	916,318	467,582	18,847	-	-	-	-	-
	OGII	1,939,015	1,681,030	1,423,045	1,165,060	907,075	649,090	391,105	133,120
	Total	2,855,333	2,148,612	1,441,892	1,165,060	907,075	649,090	391,105	133,120
2019	OGI	1,024,015	575,279	126,543	-	-	-	-	-
	OGII	1,912,700	1,654,715	1,396,730	1,138,745	880,761	622,776	364,791	106,806
	Total	2,936,715	2,229,994	1,523,273	1,138,745	880,761	622,776	364,791	106,806
Total 2017–2019		8,551,636	6,431,474	4,435,163	3,515,818	2,741,864	1,967,909	1,193,954	419,999

**Table 5:** Evolution of assumed net income in EUR, by ward occupancy from 2017 to 2019 for elective caesarean section births for a fee.

Year	Ward	The Proposed Occupancy Rate							
		85%	80%	75%	70%	65%	60%	55%	50%
2017	OGI	156,854.1	6,5871.5						
	OGII	402,661.7	350,354.4	298,047.1	245,739.7	193,432.4	141,125.1	88,817.7	36,510.4
	Total	559,515.8	416,225.9	298,047.1	245,739.7	193,432.4	141,125.1	88,817.7	36,510.4
2018	OGI	185,786.6	94,803.8	3821.3					
	OGII	393,141.9	340,834.5	288,527.2	236,219.9	183,912.5	131,605.2	79,297.9	26,990.5
	Total	578,928.4	435,638.4	292,348.5	236,219.9	183,912.5	131,605.2	79,297.9	26,990.5
2019	OGI	207,622.5	116,639.8	25,657.0					
	OGII	387,806.4	335,499.1	283,191.7	230,884.4	178,577.3	126,269.9	73,962.6	21,655.3
	Total	595,428.9	452,138.8	308,848.8	230,884.4	178,577.3	126,269.9	73,962.6	21,655.3

## Data Availability

The data presented in this study are available on request from the corresponding author.
